# Effect of Aging on ERP Components of Cognitive Control

**DOI:** 10.3389/fnagi.2016.00069

**Published:** 2016-04-06

**Authors:** Juri Kropotov, Valery Ponomarev, Ekaterina P. Tereshchenko, Andreas Müller, Lutz Jäncke

**Affiliations:** ^1^Norwegian University of Science and TechnologyTrondheim, Norway; ^2^Department of Neuropsychology, Andrzej Frycz-Modrzewski Krakow UniversityKrakow, Poland; ^3^Laboratory of Neurobiology of Action Programming, N.P. Bechtereva Institute of the Human Brain, Russian Academy of SciencesSt. Petersburg, Russia; ^4^Brain and Trauma Foundation GrisonsChur, Switzerland; ^5^Department of Psychology, Division of Neuropsychology, University of ZurichChur, Switzerland; ^6^International Normal Aging and Plasticity Research Centre (INAPIC) ZurichChur, Switzerland; ^7^University Research Priority Program “Dynamics of Healthy Aging”, University of ZurichChur, Switzerland

**Keywords:** event-related potentials, cued GO/NOGO paradigm, proactive cognitive control, latent components, blind source separation, aging, human

## Abstract

As people age, their performance on tasks requiring cognitive control often declines. Such a decline is frequently explained as either a general or specific decline in cognitive functioning with age. In the context of hypotheses suggesting a general decline, it is often proposed that processing speed generally declines with age. A further hypothesis is that an age-related compensation mechanism is associated with a specific cognitive decline. One prominent theory is the compensation hypothesis, which proposes that deteriorated functions are compensated for by higher performing functions. In this study, we used event-related potentials (ERPs) in the context of a GO/NOGO task to examine the age-related changes observed during cognitive control in a large group of healthy subjects aged between 18 and 84 years. The main question we attempted to answer was whether we could find neurophysiological support for either a general decline in processing speed or a compensation strategy. The subjects performed a relatively demanding cued GO/NOGO task with similar omissions and reaction times across the five age groups. The ERP waves of cognitive control, such as N2, P3cue and CNV, were decomposed into latent components by means of a blind source separation method. Based on this decomposition, it was possible to more precisely delineate the different neurophysiological and psychological processes involved in cognitive control. These data support the processing speed hypothesis because the latencies of all cognitive control ERP components increased with age, by 8 ms per decade for the early components (<200 ms) and by 20 ms per decade for the late components. At the same time, the compensatory hypothesis of aging was also supported, as the amplitudes of the components localized in posterior brain areas decreased with age, while those localized in the prefrontal cortical areas increased with age in order to maintain performance on this simple task at a relatively stable level.

## Introduction

There exists a consensus regarding the general pattern of age-related changes in cognition, with a nearly linear decline in episodic memory, working memory, and executive functions starting in early adulthood (Salthouse, 2010). Age-related decline on behavioral measures of cognitive control is generally related to morphological and neurophysiological changes to the prefrontal cortex with age. The particular involvement of the prefrontal cortex in aging has been emphasized in the context of several theoretical models: the frontal aging theory (Moscovitch and Winocur, 1992; West, 1996), the inhibitory deficit theory of aging (Lustig et al., 2007), and the posterior-anterior shift in aging (Davis et al., 2008).

The frontal aging theory postulates that cognitive decline in older adults is primarily due to the anatomical and functional deterioration of the frontal lobes. The inhibitory deficit theory of aging is more specific, arguing that cognitive decline in the elderly is due to a deterioration of inhibitory processes (Zacks and Hasher, 1994; Radvansky et al., 2005; Lustig et al., 2007; Darowski et al., 2008). A further hypothesis—the *processing speed hypothesis*—proposes that cognitive performance in older subjects is degraded because cognitive processing is slower and the relevant operations cannot be successfully executed (Salthouse, 1996). The posterior-anterior shift in aging theory is built on the consistent finding that age-related increases in frontal lobe activity are generally accompanied by decreased activity in the posterior brain regions (Davis et al., 2008). The posterior-anterior shift in aging is considered to be a compensatory mechanism (Cabeza et al., 2004).

Two cognitive control modes can be separated: (1) proactive and (2) reactive (Braver, 2012; Brunner et al., 2015). The *proactive mode* includes hypothetical processes such as task setting, orienting to the stimulus, attention allocation, and working memory (Braver, 2012). These processes are supposedly reflected in the following specific event-related potential (ERP) waves elicited by the cue: (1) the frontal-central N2 wave—orienting to the cue (Näätänen and Picton, 1986; Patel and Azzam, 2005); (2) the posterior late positive P3 cue—extraction of relevant information from the cue (Bekker et al., 2004); and (3) the contingent negative variation (CNV) - anticipation and motor preparation (Walter, 1967; van Boxtel et al., 2001).

Several fMRI and ERP studies have shown that reactive cognitive control include the following hypothetical operations: (1) detection of mismatch in the sensory domain (Kropotov and Ponomarev, 2015); (2) inhibition or overriding of a prepotent response pattern (Barkley, 1997; Aron, 2007); and (3) conflict detection when response tendencies are interfering with one other (van Veen and Carter, 2002; Botvinick, 2007, 2008). The cued GO/NOGO paradigm represents one of the experimental tools for studying brain operations during cognitive control (Kropotov et al., 2011; Mueller et al., 2011; Gajewski and Falkenstein, 2013; Ponomarev et al., 2014). These hypothetical operations of reactive cognitive control are reflected in ERP waves elicited in the NOGO condition, in contrast to those elicited in the GO condition, such as frontal N2 (referred here as N2d-NOGO) and frontal P3 (P3d-NOGO).

The hypothesis that the N2d-NOGO wave is associated with inhibition of action is supported by early studies showing that the N2d is larger in overt response inhibition tasks in comparison to silent counting tasks (Pfefferbaum et al., 1985; Bruin and Wijers, 2002), is increased by pressure to respond quickly (Jodo and Kayama, 1992), and is larger in participants with low false alarm rates (Falkenstein et al., 1999). However, in later studies, N2d-NOGO was evoked in GO trials when the frequency of presentations of NOGO trials was quite high (Nieuwenhuis et al., 2003), indicating that N2d-NOGO might reflect a conflict between the execution and the inhibition of a response.

Almost all studies have found age-related delays in the main ERP waves of cognitive control (Bennett et al., 2004), confirming the processing speed hypothesis of aging. With respect to the ERP amplitudes, however, the findings are less consistent. An increased CNV amplitude in old compared to young participants was found in some studies (Miyamoto et al., 1998; Hillman et al., 2002). Other studies report a reduction of CNV amplitudes in older participants (Ferrandez and Pouthas, 2001; Golob et al., 2005; Dirnberger et al., 2010), with a selective age-related reduction in CNV amplitude over the frontal areas and no change at the vertex and parietal areas (Michalewski et al., 1980; Wild-Wall et al., 2007). Some studies have reported no group differences (Bennett et al., 2004). In elderly subjects, the N2d and P3d-NOGO waves have been found to be smaller in amplitude compared to younger adults (Bennett et al., 2004), with one study reporting the opposite finding (Hong et al., 2014).

In the present article, we decompose the ERP waves of cognitive control into latent components by applying a blind source separation method (Kropotov and Ponomarev, 2015). This method is applied to a relatively large (*N* = 454) group of healthy subjects with a wide age range, from 18 to 84 years. To demonstrate gradual changes in ERP waves and their components with age, the sample is divided into five age groups: 18–23 (*N* = 114), 24–33 (*N* = 70), 34–50 (*N* = 125), 51–60 (*N* = 70), and 61–89 (*N* = 75). Two hypotheses of age-related influences on cognitive control are tested: (1) the processing speed hypothesis (Salthouse, 1996) and (2) the compensation hypothesis of aging (Cabeza, 2002; Reuter-Lorenz and Cappell, 2008).

## Materials and Methods

### Subjects

Four hundred fifty four healthy subjects (age range: 18–89) participated in the study. Subjects were recruited from Chur (Switzerland) during the database collection project headed by Dr. Andreas Müller, from students of St. Petersburg State University and staff of the Institute of the Human Brain of Russian Academy of Sciences. All subjects were free of obvious neurological and psychiatric problems, demonstrated at least average cognitive skills, no vision or hearing problems. Subjects suffering from neurological or systemic medical diseases were excluded from the study. Subjects had to score lower than the level of clinical significance on a symptom checklist (Franke, 2000). Most of the subjects were right-handed by self-report (17 left-handed, 10 ambidexter). For the older subjects we carefully checked whether they demonstrate signs of mild cognitive impairment or even dementia by using a standard cognitive test. The investigation was carried out in accordance with the Declaration of Helsinki, and all subjects provided informed consent. The local ethics committee of the canton Graubunden, district of Grison (Switzerland) approved this study (EK 20/07).

### Construction of Trials

To study brain correlates of cognitive control a specific variant of the cued GO/NOGO task was used (Kropotov and Ponomarev, 2009; Kropotov et al., 2011). In this task, images of animal (*a*) and plant (*p*) categories served as relevant stimuli. This task has been described in the above-mentioned studies extensively. Thus, we will describe them here shortly by paraphrasing what has been said in the above-mentioned articles. The trials consisted of the presentations of paired stimuli ***s1***-***s2*** with inter-stimulus intervals of 1000 ms and inter-trial intervals of 3000 ms. Four categories of trials were used: *a-a, a-p, p-p and p-h + novel sound*, where *h* is an image of a human. The duration of stimuli was 100 ms. The subject’s task was to respond to a-a trials so that the trials starting with the presentation of *p* (referred to in this article as NonCue) at the first position indicated no further action in the trial while trials starting with the presentation of *a* (referred to in this article as Cue) at the first position required preparation for receiving the next stimulus and pressing a button in response to the same *a* stimulus or withholding from action in response to *p*. The speed and accuracy were equally emphasized. In the present article, the responses to Cue and NonCue up to the second stimulus are computed and contrasted with each other.

The pictures were selected from textbooks for children in such a way that the overall luminance and the image sizes of animals and plants were approximately equal. To avoid habituation to repeating stimuli, 20 different images of animals, plants and humans were randomly presented in various combinations. To maintain a certain level of alertness, novel sounds were occasionally presented simultaneously with the images of a human in ignore trials. They produced an orientation reaction, confirmed by the elicitation of the P3 novelty ERP wave.

The trials were grouped into four blocks with one hundred trials each. In each block a unique set of five *a*, five *p*, and five *h* stimuli were selected. Each block consisted of a pseudo-random presentation (requiring equal number of trials in four categories) of 400 trials with 100 trials within each trial category. Participants practiced the task before the recording started. Subjects rested for a few minutes after each 200 trials. Subjects sat upright in a comfortable chair looking at a computer screen. Stimuli were presented on 17” CRT computer screens which were positioned 1.5 m in front of the subjects and occupied 3.8° of the visual field. All trials were presented using *Psytask*, a computer code written by one of the authors (Ponomarev).

### Data Recording

The subject responses (by right hands) were recorded in a separate channel. An average for response latency was calculated for each subject individually. Omission errors (failure to respond in GO trials) and commission errors (failure to suppress a response to NOGO trials) were also computed for each subject separately. EEG was recorded from 19 scalp sites according to the 10–20-system, bandpass-filtered between 0.3 and 50 Hz, and digitized at a rate of 250 samples per second per channel.

The EEG was recorded referentially to linked ears, allowing computational re-referencing of the data (re-montaging). For decomposing ERPs into independent components, the EEG computationally was re-referenced to the common average montage. EEG was recorded with a 19-channel electroencephalographic PC-controlled system, the “Mitsar-201” (CE 0537) manufactured by Mitsar Co., Ltd, Russia. Electrodes were applied using caps manufactured by Electro-Cap International, Inc, OH, USA. The tin recessed electrodes contacted the scalp using ECI ELECTRO-GEL. Quantitative data were obtained using WinEEG Software (Ponomarev and Kropotov, 2013). Eye blink artifacts were corrected by zeroing the activation curves of individual independent components corresponding to eye blinks. These components were obtained by application of *Independent Component Analysis* (ICA) to the raw EEG fragments. The method is described in Jung et al. (Vigário, 1997; Jung et al., 2000a,b). Comparison of the method applied in our study with an EOG regression technique is described in Tereshchenko et al. (2009). In addition, epochs with excessive amplitude of filtered EEG and/or excessive faster and/or slower frequency activity were automatically marked and excluded from further analysis. The exclusion thresholds were set as follows: (1) 100 μV for non-filtered EEG; (2) 50 μV for slow waves in 0–1 Hz band; and (3) 35 μV for fast waves filtered in the band 20–35 Hz.

### Decomposition of Collection of ERPs into Latent Components

The event related potential $x_{i}^{j}(t)$ at *i*-electrode (*i* = 1, …, M) of *j*-subject (*j* = 1, …, P) at time point *t* (*t* = 1, …, T) is modeled as a linear combination of hidden (latent) signals $s_{k}^{j}(t)$, *K* ≤ *M*, with some unknown coefficients *a_ik_*,

$$x_{i}^{j}(t)\;=\;\sum_{k\;=\;1}^{K}a_{i,k}s_{k}^{j}(t)+\varepsilon_{i}^{j}(t)$$

where ε is the noise component that is assumed to be additive, stationary, isotropic and independent of the signals $s_{k}^{j}(t)$. *A* is the *M* × *K* mixing matrix with elements *a_ik_*, which is assumed to be the same for all subjects. To separate the latent components hidden in ERPs we used a method of blind source separation based on second-order statistics (see Yeredor, 2010) and adapted for the transient responses (Ponomarev and Kropotov, 2013). The main assumption underlying the separation method is that the variability of the deviations of individual ERPs from the group average potentials can be modeled by linear combinations of low correlated signals (Ponomarev and Kropotov, 2013; Kropotov and Ponomarev, 2015).

To estimate the elements *a_ik_* of the mixing matrix *A* of the proactive cognitive control we used the large collection of ERPs computed for Cue (***s1*** = animal) and NonCue (***s1*** = plant) trials in the interval (*T* = 1100 ms) between ***s1*** and ***s2***. To estimate *a_ik_* of the mixing matrix *A* of the reactive cognitive control we used the large collection of ERPs computed for GO (***s1*** = animal, ***s*** = the same animal) and NOGO (***s1*** = animal, ***s2*** = plant) trials in the interval (*T* = 1100 ms) after ***s2***.

To determine the optimal number of hidden signals (latent components) *K* we used a model selection method based on the Bayesian information criterion (BIC; MacKay, 1992; Hansen et al., 2001; Kolenda et al., 2001). This approach was adapted for the transient responses with a detailed description of this method presented in Kropotov and Ponomarev (2015). The split-half reliability of the model was estimated by repeating the procedure 1000 times for a randomly selected half of the total ERP set (Kropotov and Ponomarev, 2015).

### Imaging of Latent Components

sLORETA imaging approach was used for locating the generators of the ICA components. The free software is provided by the Key Institute for Brain-Mind Research in Zurich, Switzerland. For theoretical issues of this “Materials and Methods” Section, see (Strik et al., 1998; Pascual-Marqui, 2002). sLORETA provides images of current density with exact localization, albeit with low spatial resolution. Here, we have used the version from 2002 which offers a solution space restricted to the cortical gray matter, corresponding to 2394 voxels at a 7-mm spatial resolution. Several articles have demonstrated the validity of LORETA intracortical estimations (Vitacco et al., 2002; Zumsteg et al., 2005, 2006). We are aware of the fact that the validity of the LORETA estimation strongly depends on the number of electrodes used, with more electrodes (mostly) providing better estimations. However, we would like to point out that we explicitly used a 19-electrode EEG montage here in order to make the EEG registration as nonintrusive as possible for the subjects. In addition, we were not interested in a perfect intracortical solution; rather, we were interested in obtaining a more or less rough imaging of the generators of independent latent components. Nevertheless, we would like to emphasize that intracortical estimations on the basis of 19-electrode EEGs can indeed provide valid results, as has been shown in several studies (Zumsteg et al., 2005; Emory et al., 2015), especially when using clean EEG data without artifacts. In the current study, we estimated the intracortical sources on the basis of mean independent components, thus diminishing the influence of artifacts.

### Statistical Analysis

#### Analysis of Behavioral Data

Omission, commission errors and reaction times were assessed using analysis of variance (one-way ANOVA, factor with two levels). The response was considered correct if it was made to the appropriate stimulus and started in the time interval from 100 to 1000 ms.

#### Analysis of the ERP

Before performing statistical assessment of the latent components a preliminary analysis of ERPs was made in order to show that changing the experimental condition indeed changed the ERP waves. A two-way repeated measures ANOVA with the following factors was used: Age (non-repeated) and Location (repeated). This type of analysis was performed both for the ERPs, and for the latent components. Finally, an additional analysis was carried out for the latent components only. In this case, the values of difference waves in the selected time interval were compared between age groups. Intervals were chosen for each latent component separately on the basis of local peaks of the difference curves. In this way, the onset of offset of the selected time interval corresponded approximately to half maximum of the peak value of the difference. Further, the average values of the difference waves in the selected time interval were calculated for each subject, and the corresponding mean values, standard deviations and 95% confidence intervals were obtained for each experiment separately.

## Results

### Behavioral Data

Table 1 shows descriptive statistics of subjects’ behavior for each trial type and each age group. Note that the mean percent of omission and commission errors was below 2.5% in all age groups and the mean reaction time remained practically the same and did not-systematically vary between 390 and 410 ms for the different age groups. Thus, although the behavioral data are roughly similar the ERP differences reported below cannot be attributed to different behavioral performances.

**Table 1:** **Mean ± SD of behavioral performance measures broken down for the different age groups**.

Age group	Number of subjects (males)	GO omissions	NOGO false alarms	RT in GO trials (ms)	Error of variance of RT
18–23	114 (50)	1.60 ± 2.38	0.82 ± 1.46	407 ± 88	9.02 ± 2.69
24–33	70 (39)	1.20 ± 1.61	0.64 ± 1.02	385 ± 88	7.84 ± 2.73
34–50	125 (48)	1.47 ± 2.45	0.60 ± 1.16	398 ± 83	7.55 ± 2.58
51–60	70 (24)	1.62 ± 2.26	1.03 ± 2.28	391 ± 80	7.17 ± 2.62
61–89	75 (31)	2.33 ± 2.81	1.61 ± 2.36	389 ± 68	7.31 ± 2.80

### Grand Average ERPs

#### ERPs to Stimulus 1 (*s1*)

**Figure 1:**
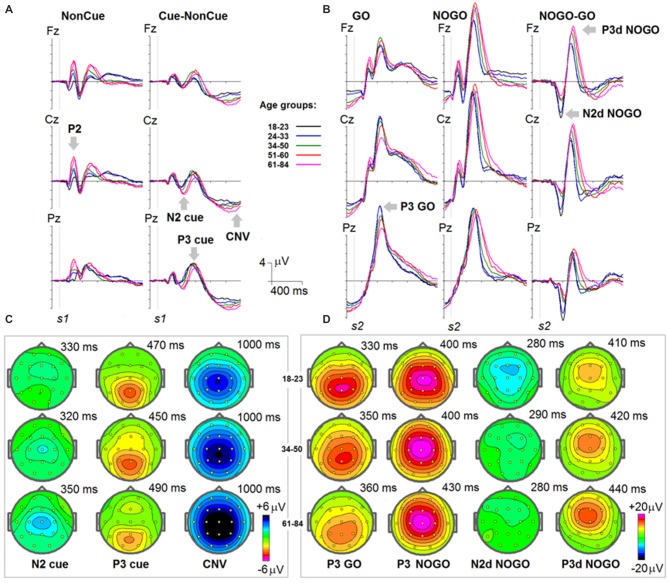
**Age dynamics of event-related potentials (ERPs) in the cued GO/NOGO task.** In the task, images of animals and plants are presented in different *s1-s2* pairs with the task to press a button as fast and precise as possible to “animal-animal” pairs. **(A)** ERPs in response to *s1* for NonCue (where NonCue is an image of plant at the first position in the trial) and ERP differences Cue-NonCue (where Cue an image of animal at the first position of the trails) for different age groups (18–23: black, 24–33: blue, 34–40: green, 51–60: red and 61–84: pink colors) and for Fz, Cz and Pz. *X*-axis time in ms. *Y*-axis—voltage in μV. The waves P2, N2 cue, P3 cue and CNV are marked by arrows. **(B)** Maps of the N2, P3 cue and CNV waves for three age groups (18–23, 34–40 and 61–84). **(C)** ERPs in response to *s2* for GO (animal-animal) pairs, NOGO (animal-plant) pairs and the difference NOGO-GO waves for different age groups (18–23: black, 24–33: blue, 34–40: green, 51–60: red and 61–84: pink colors) and for Fz, Cz and Pz. *X*-axis time in ms. *Y*-axis—voltage in μV. The waves P3 GO, N2d NOGO and P3d NOGO are marked by arrows. **(C)** Maps of the P3 GO, N2d NOGO and P3d NOGO waves for three age groups (18–23, 34–40 and 61–84).

Grand average ERPs to NonCue as well as the difference waves Cue-NonCue for the five age groups are presented in Figure 1A. A central-frontal P2 wave is elicited in response to the NonCue whereas no prominent central-parietal CNV waves are observed. The difference waves Cue-NonCue show that the Cue in contrast to the NonCue elicits the central N2 and parietal P3 waves. Maps at peak amplitudes of N2Cue and P3Cue waves are presented at the bottom of Figure 1A. Peak latencies are presented at the right of the maps in Figure 1. The results of statistical analysis are presented in Table 2. As one can see from the Figure and the Table, the amplitudes of the centrally distributed N2Cue and CNV increase with age, while the amplitude of the parietally distributed P3Cue shows an opposite pattern. The peak latencies of both N2Cue and P3Cue increase by age with approximately 4 ms per decade.

**Table 2:** **Statistical analysis of amplitudes of the waves in NonCue condition and in the difference Cue–NonCue**.

Wave	Interval (ms)	Age effect *F*_[4, 449]_, *p* <	Age/electrode interaction *F*_[72, 8082]_, ε, *p* <
**NonCue**
P1	100–130	4.85, 10^−3^	3.48, 0.11, 10^−3^
N1	130–190	24.5, 10^−7^	10.3, 0.09, 10^−6^
P2	140–240	33.5, 10^−7^	17.0, 0.09, 10^−7^
CNV	800–1100	9.59, 10^−6^	13.7, 0.15, 10^−6^
**Cue—NonCue**
P1	100–130	2.0, NS	2.85, 0.22, 10^−3^
N1	130–190	0.86, NS	2.45, 0.23, 0.01
N2Cue	280–390	4.19, 0.01	13.7, 0.21, 10^−7^
P3Cue	390–530	4.81, 10^−3^	6.45, 0.20, 10^−6^
CNV	800–1100	19.2, 10^−6^	4.72, 0.20, 10^−6^

#### ERPs to Stimulus 2 (*s2*)

Grand average ERPs for the GO, NOGO stimuli as well for the difference waves NOGO-GO for the five age groups are presented in Figure 1B. A parietally distributed P3Go wave is elicited in response to the GO stimulus at peak latencies between 330 and 360 ms. The NOGO stimulus in contrast to the GO stimulus elicits the frontal N2d NoGo wave with peak latency of about 280 ms measured at Fz and the frontal P3d NoGo wave with peak latency of 410–440 ms. Maps at peak amplitudes of these waves for the five age groups are presented at the bottom of Figure 1B. Peak latencies are presented at the right of the maps. The results of statistical analysis are presented in Table 3. As one can see in Figure 1B, the parietally distributed P3Go wave decreases with age. The amplitude of the frontally distributed N2d NOGO decreases with age while the amplitude of the P3d NOGO wave shows opposite age dynamics. The peak latencies of the positive waves increase by age with approximately 6 ms per decade whereas the peak latency of N2d NOGO measured at Fz remains stable.

**Table 3:** **Statistical analysis of amplitudes of the waves in the Go and NoGo conditions, and for the difference between NoGo–Go**.

Wave	Interval (ms)	Age effect *F*_[4, 449]_, *p* <	Age/electrode interaction *F*_[72, 8082]_, ε, *p* <
**Go**
P1	110–130	0.85, NS	3.52, 0.14, 10^−3^
N1	140–190	2.08, NS	5.47, 0.11, 10^−6^
P2	180–250	1.93, NS	14.7, 0.14, 10^−7^
P3Go	240–380	5.491, 10^−3^	8.43, 0.19, 10^−7^
**NoGo**
P1	110–130	0.12, NS	3.36, 0.15, 10^−3^
N1	140–190	3.30, NS	6.68, 0.11, 10^−6^
P2	200–260	3.14, NS	22.9, 0.12, 10^−7^
N2	200–280	3.85, 0.01	18.1, 0.13, 10^−7^
P3NoGo	320–410	4.26, 0.01	13.4, 0.19, 10^−7^
	440–620	52.8, 10–7	18.4, 0.20, 10^−7^
**NoGo-Go**
N2dNoGo	220–300	19.3, 10^−7^	11.8, 0.22, 10^−7^
P3dNoGo	360–420	1.86, NS	14.4, 0.21, 10^−7^

### Decomposition of Group ERPs into Latent Components

**Figure 2:**
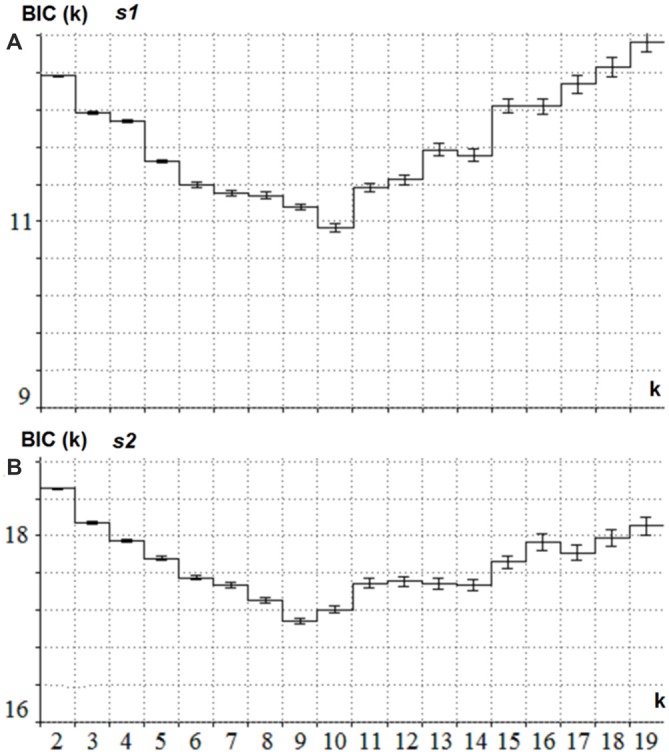
**Estimation of the optimal number of the latent components for *s1* and *s2*. (A)** *BIC* (*Y*-axis) as a function of the number of components k (*X* -axis) for the collection of ERPs in Cue and NonCue conditions and time interval between *s1* and *s2*. The minimum of the curve corresponds to the optimal number of latent components. **(B)** *BIC* (*Y*-axis) as a function of the number of components k (*X* -axis) for the collection of ERPs in GO and NOGO conditions and 1.1 s time interval after *s2*. The minimum of the curve corresponds to the optimal number of latent components.

The latent components were separately computed for ***s1*** and ***s2***. For ***s1*** the joint collection of ERPs for the Cue and NonCue conditions in the time interval of 1100 ms after onset of ***s1*** was used. For ***s2*** the joint collection of ERPs to GO and NOGO conditions in the time interval of 1100 ms after onset of ***s2*** was used. The BIC as a function of a number of latent components (k) is presented in Figure 2 separately for ***s1*** and ***s2***. This minimum corresponds to the optimal number of the components for modeling the ERPs.

**Figure 3:**
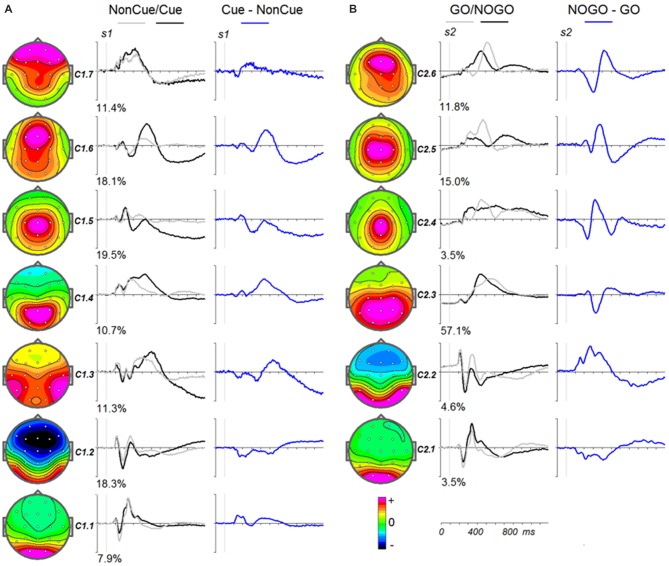
**Modulation of the latent components in the cued GO/NOGO task by operations of proactive and reactive cognitive control in the average age group (34–40 years old). (A)** Latent components in response to *s1* for Cue (black), NonCue (gray) and differences Cue-NonCue (blue). Left—topographies of the components. **(B)** Latent components in response to *s2* for GO (black), NOGO (gray) and differences NOGO-GO (blue). Left—topographies of the components.

To illustrate the latent components related to NonCue and the modulations induced by Cue (Cue-NonCue differences) Figure 3A shows the corresponding curves for the middle age group (34–50 years old). To illustrate the latent components related to GO stimulus and the modulations induced by NOGO stimulus (NOGO-GO differences). Figure 3B shows the corresponding curves for the middle age group (34–50 years old).

The parameters of split-half reliability of the seven estimated components for ***s1*** and six estimated component for ***s2*** are presented in Table 4. The topographies and time courses of other components haven’t been reliably estimated and are not shown. The presented in Figure 3 components explain more than 90% of the signal variance. T Localization of the latent components according to sLORETA is presented in Table 5.

**Table 4:** **Split-half reliability and relative power of the latent components**.

Component	ř_T_ ± *σ_T_*	ř_ERP_ ± *σ_ERP_*
***s1*, Cue and NonCue conditions**
C1.7	0.97 ± 0.07	0.87 ± 0.21
C1.6	0.91 ± 0.15	0.80 ± 0.30
C1.5	0.97 ± 0.04	0.91 ± 0.11
C1.4	0.92 ± 0.10	0.91 ± 0.20
C1.3	0.94 ± 0.10	0.94 ± 0.14
C1.2	0.97 ± 0.07	0.86 ± 0.22
C1.1	0.99 ± 0.02	0.96 ± 0.04
***s2*, Go and NoGo conditions**
C2.6	0.98 ± 0.04	0.94 ± 0.10
C2.5	0.98 ± 0.02	0.92 ± 0.09
C2.4	0.90 ± 0.16	0.80 ± 0.21
C2.3	0.98 ± 0.03	0.97 ± 0.06
C2.2	0.97 ± 0.04	0.97 ± 0.06
C2.1	0.97 ± 0.04	0.97 ± 0.05

*ř_T_ and σ_T_-similarity index and standard deviation its for the topographies. ř_ERP_ and σ_ERP_ -similarity index and standard deviation for the time course*.

**Table 5:** **Localization of the latent components according to sLORETA**.

Component	Gyrus	BA
***s1*, Cue and NonCue conditions**
C1.7	Uncus	20
C1.6	Medial Frontal Gyrus,	11
	Rectal Gyrus	
C1.5	Superior Frontal Gyrus	8
C1.4	Paracentral Lobule	5
C1.3	Superior Temporal Gyrus	42R
C1.2	Fusiform Gyrus,	20R, 36R
	Parahippocampal Gyrus	
C1.1	Cuneus	19
***s2*, Go and NoGo conditions**
C2.6	Superior Frontal Gyrus,	10, 11
	Medial Frontal Gyrus	
C2.5	Cingulate Gyrus	23
C2.4	Postcentral Gyrus	3
C2.3	Medial Frontal Gyrus	6
C2.2	Fusiform Gyrus	20R, 36R
C2.1	Cuneus, Precuneus	19, 31

### Age Dynamics of Latent Components

#### s1

**Figure 4:**
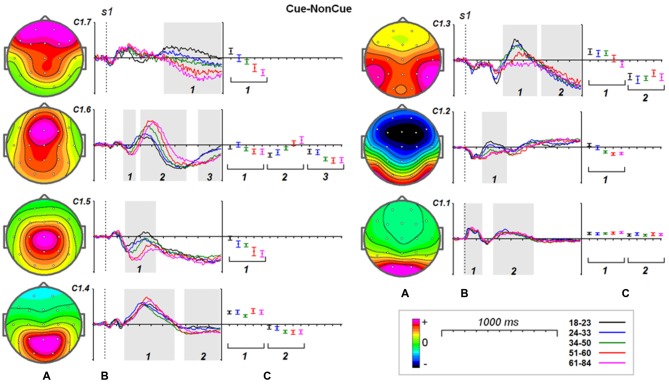
**Age dynamics of the modulation effect of the proactive cognitive control on the latent components in response to *s1*. (A)** Topographies of latent components. **(B)** Cue-NonCue differences for five age groups (18–23: black, 24–33: blue, 34–40: green, 51–60: red and 61–84: pink colors). Gray areas define time windows (1, 2, 3) for which the statistical analysis was performed. Dashed vertical line—*s1* presentation. **(C)** Mean values and 95% confidence intervals computed for the time windows for five age groups.

Age dynamics of the modulation of latent components by the Cue are presented in Figure 4. Statistical significance of the age effects is presented in Table 6. The two latent components C1.6 and C1.5 represent decomposition of the N2Cue wave. The amplitude of both components measured in the corresponding time interval decrease with age—the same pattern as observed for the N2-Cue wave.

**Table 6:** **Statistical analysis the latent components extracted for *s1* and computed for NoCue condition and for Cue-NonCue difference**.

	NoCue			Cue-NonCue		
Component	Interval, ms	*F*_[4, 449]_	*p* <	Interval, ms	*F*_[4, 449]_	*p* <
C1.7	510–740	3.35	NS	600–1100	31.1	10^−6^
C1.6	340–640	15.8	10^−6^	250–360	5.87	10^−3^
				400–800	26.5	10^−6^
				900–1100	5.6	10^−3^
C1.5	180–260	6.16	10^−4^	270–540	15.4	10^−6^
	350–560	10.4	10^−6^			
C1.4	220–520	10.4	10^−6^	270–700	4.8	10^−3^
				790–1100	6.45	10^−4^
C1.3	300–530	15.3	10^−6^	420–720	12.5	10^−6^
				760–1100	2.09	NS
C1.2	140–220	41.5	10^−7^	250–460	30	10^−7^
	240–360	11.6	10^−6^		
	560–830	42.5	10^−7^		
C1.1	120–180	1.66	NS	90–250	0.56	NS
	210–360	7.28	10^−4^	340–700	0.7	NS

A more complex pattern of age dynamics is found for three components loaded into P3-Cue wave. The components have distinct parietal, temporal and frontal distributions and change with age in a different way: the amplitude of the temporal component dramatically decreases with age, the amplitude of the parietal components does not change with age, whereas amplitude of the frontal component increases with age.

As one can see from Figure 4 all of the components contribute to CNV wave but with different weights. The largest contribution is made by the central component C1.5 and temporal component C1.3. The negative part of the components preceding ***s2*** doesn’t change with age. In the same time the frontal components C1.7 and C1.6 increase negative amplitude preceding ***s2***.

It should be stressed here that beside the proactive cognitive control components the decomposition includes two components C1.1 and C1.2 that don’t contribute to the ERP waves associated with cognitive control. The C1.1 component is generated in the precuneus while the C1.2 component is generated in the inferior temporal cortex. These components are modulated by task setting (Cue vs. NonCue) as early as 110 ms after stimulus. The modulations of the occipitally generated component don’t change with age in the whole time interval, while the modulations of the temporal component at time interval of 250–400 ms change with age.

Increase in timing of the modulation of the sensory-related components C1.1 and C1.2 changes with age on average about 4 ms per decade whereas latencies of the P3Cue-related components increase with age on average about 20 ms per decade.

#### s2

**Figure 5:**
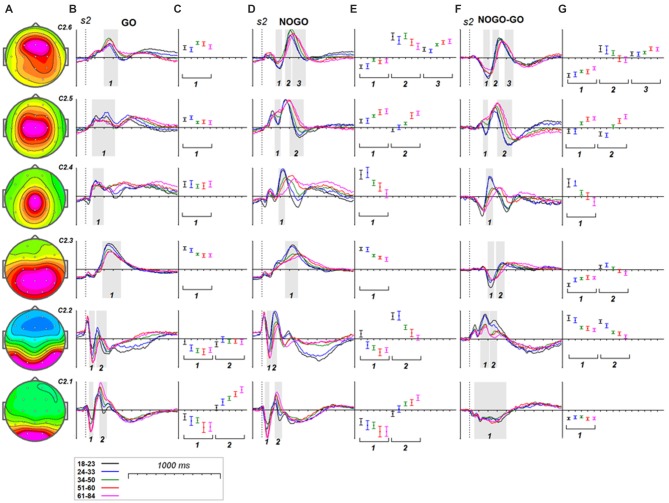
**Age dynamics of the modulation effect of the reactive cognitive control on the latent components in response to *s2*. (A)** Topographies of latent components. **(B)** latent components for GO condition for five age groups (18–23: black, 24–33: blue, 34–40: green, 51–60: red and 61–84: pink colors). Gray areas define time windows (1, 2, 3) for which the statistical analysis was performed. Dashed vertical line—***s2*** presentation. **(C)** Mean values and 95% confidence intervals computed for the time windows for five age groups. **(D,E)** as above for **(B,C)** for the latent components for NOGO condition. **(F,G)** as above for **(B,C)** for the difference NOGO-GO of latent components.

Age dynamics of the GO components and their modulation by NOGO conditions are presented in Figure 5. Statistical significance of the age effects is presented in Table 7. The P3Go wave is decomposed into two components with the parietal one showing decrease with age and the frontal one showing increase with age. The three components are loaded into the N2d and P3d NoGo wave. For all of these components the parts loaded into the N2 wave decrease with age whereas the parts loaded into the P3 NOGO wave behave differently depending on localization: increase for the centrally distributed component, don’t change in amplitude for the frontally distributed component, and dramatically decrease with age for the parietally distributed component.

**Table 7:** **Statistical analysis the latent components extracted for *s2* and computed for Go and NoGo conditions and for NoGo-Go difference**.

	Go			NoGo			NoGo-Go		
Component	Interval, ms	*F*_[4,449]_	*p* <	Interval, ms	*F*_[4,449]_	*p* <	Interval, ms	*F*_[4,449]_	*p* <
C2.6	300–450	7.42	10^−5^	250–320	11.9	10^−6^	250–320	9.28	10^−6^
				350–420	6.74	10^−4^	350–420	9.64	10^−6^
				420–580	17.3	10^−6^	480–580	6.92	10^−4^
C2.5	170–420	3.03	NS	240–300	18.7	10^−6^	240–300	36.1	10^−6^
				400–560	44.7	10^−7^	400–560	55.9	10^−6^
C2.4	180–300	0.63	NS	280–350	16.7	10^−6^	280–350	21.1	10^−6^
C2.3	280–480	18.3	10^−6^	350–500	28	10^−7^	300–370	15.2	10^−6^
							390–480	15	10^−6^
C2.2	140–200	9.94	10^−6^	150–200	23.9	10^−7^	220–300	21.3	10^−6^
	220–320	2.42	NS	200–260	37.3	10^−7^	300–400	52.1	10^−7^
C2.1	140–180	5.86	10^−3^	130–180	4.19	0.01	150–500	1.57	NS
	250–330	30.1	10^−7^	240–320	23.6	10^−6^		

## Discussion

### Behavior

The behavioral performance is roughly similar for the different age groups so that anticipated neurophysiological differences cannot be attributed to different performance levels.

### ERP Components of Proactive Cognitive Control

The proactive mode includes hypothetical processes such as orienting to stimuli, attention allocation, and working memory (Braver, 2012) are reflected in ERP waves: (1) the frontal-central N2 Cue—orientation to cue (Patel and Azzam, 2005); (2) the posterior late positive P3 cue—extraction of relevant information from cue (Bekker et al., 2004); and (3) the contingent negative variation (CNV)—anticipation and motor preparation (Walter, 1967; van Boxtel et al., 2001). The N2, P3 and CNV waves observed in this study are similar to those obtained in different variants of the cued task (Gratton et al., 1990; Bekker et al., 2004; Galvao-Carmona et al., 2014). Our ERP data show age-related decrease in the parietal P3 cue accompanied by increased of N2 Cue and CNV waves. Our data fit with the results of Hong et al. (2014) and suggest that the process of extracting relevant information from the cue decreases with age, while the processes of orientation and preparation elicited by the cue increase with age (Cabeza, 2002; Reuter-Lorenz and Cappell, 2008).

#### Attention to the Cue

As our data show demonstrate that the frontal N2 wave is decomposed into the two components, C1.6 and C1.5, generated in the medial frontal gyrus (MFG) and in the superior frontal gyrus (SFG), respectively. The latency of the MFG N2 is around 300 ms and shows no change with age. The latency of the SFG N2 substantially increases with age, by around 20 ms per decade. N2 amplitudes for both components, however, increase with age. Both N2 fluctuations are followed by late positive fluctuations, which change with age quite differently: (1) the C1.6 component increases in amplitude and latency with age, and (2) the C1.5 component decreases in amplitude with age. These data suggest that the MFG, in contrast to SFG, becomes more reactive to the cue with increasing age.

#### Extraction of Relevant Information from the Cue

The components C1.3 and C1.4 contribute to the P3 cue wave elicited by the cue. The components are generated in the paracentral lobule and superior temporal gyrus. The presentation of the cue elicits a late positivity around 450 ms in the parietal cortex and around 530 ms in the temporal cortex. The peak latency of positive fluctuations for both components increases with age, while the magnitude remains stable with age for the parietal component and decreases with age for the temporal component. This result indicates that the temporal areas, in comparison to the parietal areas, are more vulnerable to structural changes accumulating with age, similar to the findings of others (Salat, 2011).

#### Preparation for the Target

Our ERP data fit with the results of another study showing an increased frontal CNV amplitude in older participants (Wild-Wall et al., 2007). All latent components extracted in our study, except C1.1, show slow shifts preceding ***s2*** presentation and can be associated with the expectancy CNV wave (Brunia and van Boxtel, 2001). The negative wave of C1.6 generated in the MFG with peak latency at around 700 ms can be associated with the frontally-localized orienting CNV (Brunia and van Boxtel, 2001). Our data show that the age dynamics of expectancy potentials depend on their localization: the components C1.7 and C1.6, generated in the medial frontal-temporal areas, show a substantial increase in amplitude with age, while the components generated in superior temporal and superior frontal areas, C1.3 and C1.5 do not change significantly with age.

### ERP Components of Reactive Cognitive Control

In the cued GO/NOGO task used in this study, the hypothetical processes of proactive cognitive control include context updating, detection of the mismatch sensory and action domains, inhibition or overriding of action and monitoring of the situation (Folstein and Van Petten, 2008; van Noordt and Segalowitz, 2012). Context updating is associated with the parietally distributed P3 GO wave (Adrover-Roig and Barceló, 2010), while the detection of mismatch in sensory domain is associated with the temporally distributed P2 wave (Kropotov and Ponomarev, 2015). Conflict detection is associated with the frontal N2d NOGO wave, whereas inhibition of action is associated with the frontal P3d NOGO wave (Smith et al., 2008; Enriquez-Geppert et al., 2010; Albert et al., 2013). Our data showing decreased N2d with aging fit with the results of Hämmerer et al. (2010) and our findings of increased P3d with aging fit with the results of Hong et al. (2014). Decomposing the ERP waves of reactive cognitive control into latent components revealed the following pattern.

#### Comparison to Working Memory and Action Conflict Detection

At least two latent components contributed to the frontal N2d NOGO wave. They are component C2.2, generated in the fusiform gyrus, and component C2.6, generated in the superior frontal and/or MFG. Both components decrease in amplitude with age. The first component reflects the hypothetical operation of comparison to working memory whereas the second component reflects detection of conflict between different behavioral options (GO vs. NOGO; Kropotov et al., 2011; Kropotov and Ponomarev, 2015). Consequently, we can speculate that the brain operations of detecting different types of conflict decline with age. It should be noted here that the N2 fluctuation of the C2.6 component is followed by the P3 fluctuation. This activity does not decay in amplitude with age but increases in latency. We speculate that this activity is functionally different from the frontal N2-like activities and might be associated with process of conflict resolving, i.e., a brain adjustment to avoid conflict in the future after detecting the current conflict. It appears that the power of this operation does not decline with age.

#### Inhibiting Prepotent Response

The P3-like activity of component C2.4 appears to be associated with the overriding of the prepotent response (Kropotov et al., 2011; Brunner et al., 2013, 2015; Kropotov and Ponomarev, 2015). Furthermore, the P3-like fluctuation for the difference wave was shown to appear in conditions in which the subject needed to suppress the prepared action and disappear when the subject needed to respond to match and mismatch trials with different hands (Kropotov et al., 2011). As the present data show, the P3-activation of this component dramatically declines in amplitude and latency with age. This corresponds to the hypothesis of an inhibition deficit in the elderly (Lustig et al., 2007).

### Sensory Related ERP Components

Although this article’s focus is on the components of cognitive control, we cannot avoid discussing the findings for the sensory-related components. These are components C1.1, C1.2, C2.1 and C2.2. The components C1.1 and C2.1 show occipital distribution, with localization in the precuneus, while components C1.2 and C2.2 are localized in the fusiform gyrus. Our data show that amplitude of N1-like parts of the components increase with age. This finding corresponds to previous studies showing enhanced posterior N1 in the elderly population (Yordanova et al., 2004). Previous studies also showed that the posterior N1 amplitude correlates with inter-individual differences in processing speed and reflects the amount of available neuronal resources for visual processing, so that faster individuals need to recruit a relatively smaller amount of their available resources when categorizing objects (Wiegand et al., 2014). We can further speculate that enhancement of N1 in our latent components indicates a compensatory mechanism, according to which elderly people engage more resources to complete the simple discrimination task. This suggestion fits with fMRI studies (Vallesi et al., 2011). Modulation of the sensory-related components by the task requirements during the first 200 ms is associated with the category discrimination operation (Kropotov and Ponomarev, 2015). This modulation does not change with age for the occipital component and decreases with age for the temporal component. The increase in latency for the Cue-NonCue difference with age is very small, at about 5 ms per decade. This is consistent with the delay rate found in the face discrimination task (Rousselet et al., 2010).

On the basis of experimental evidence obtained in the present study, we can conclude that the activation patterns and the category discrimination modulatory effect change differentially with age for the different time intervals and different components (i.e., cortical locations): the less dramatic changes are observed in the striate cortex, whereas the temporal cortex shows quite large changes, especially at the late (around 200 ms) latencies.

### Processing Speed Hypothesis

Our data unequivocally support the processing speed hypothesis of aging (Salthouse, 1996). Two observations from our data should be mentioned with respect to this hypothesis. First, increase in latency for the cognitive control components does not depend on changes in amplitude. Second, increase in latency for a component depends on its peak latency: the increase is relatively small (around 5–6 ms per decade) at shorter (100–200 ms) latencies and almost twice as large (around 16 ms per decade) at longer (400–500 ms) peak latencies. If we consider that the peak of a component is defined by the efficiency of information flow in the corresponding network, we may speculate that the speed delay in the elderly is due to damage to the myelin sheath and reduction in the total number of nerve fibers found in elderly people (Salat, 2011). However, although such structural damage may explain the reduction in amplitude and increase in latency for the parietally distributed P3 GO, it cannot directly explain the enhancement of the frontally distributed P3 NOGO wave. To explain this finding, we need to admit that increase in the frontal lobe component in elderly plays a compensatory role (Cabeza, 2002; Reuter-Lorenz and Cappell, 2008).

### Compensation Hypothesis of Aging

Our data fit with the compensation hypothesis of aging, which states that older adults cognitively compensate for loss of function due to neuroanatomical degeneration (Park and Reuter-Lorenz, 2009). Indeed, for proactive cognitive control, reduction of the P3cue in component C1.3 (generated in the superior temporal cortex) is accompanied by an increased P3 in component C1.6 (generated in the MFG). Similarly, for reactive cognitive control, the decrease in component C2.2 generated in the fusiform gyrus is accompanied by an increase in component C2.5 generated in the cingulate cortex. Our ERP data support the recent finding of a conjunction between dorsolateral prefrontal cortex gray matter reduction and functional MRI hyperactivation in cognitive tasks (Di et al., 2013).

### Posterior-Anterior Shift in Aging

The ERP results in our study show that the parietally distributed P3 cue wave decreases in amplitude with age, at approximately 0.2 μV per decade, while the centro-frontally distributed N2 wave increases with age, at approximately 0.4 μV per decade. These results are consistent with recent studies on proactive control in aging (Hämmerer et al., 2010; Kopp et al., 2014) as well as with the numerous findings of age-related P3b in the auditory oddball paradigm (for a recent review, see Rossini et al., 2007). If we associate the amplitude of the component with the amount of neuronal resources used for the specific associated operations associated, we can speculate that the age-related decline in the process of extracting task-related information from the relevant stimulus is accompanied by increased orientation to that stimulus. This speculation fits with the results of a recent fMRI study which revealed that age-related reduction in occipital-temporal-parietal activity is coupled with age-related increase in frontal activity, the pattern called the posterior-anterior shift in aging (Davis et al., 2008). The progressive age-dependent anteriorization of topographies of the P3 NonCue, P3 Cue and CNV waves found in our study is similar to anteriorization of P3b topography in aging (Friedman, 2013) and represents the ERP counterpart of the posterior-anterior shift in aging.

### Limitations of the Study and Further Directions

It should be noted here that several methods using blind source separation have been applied in ERP research (e.g., Delorme et al., 2012). Each of these methods relies on some general assumptions about the nature of ERP signals. In this article, we use an approach that enables us to not only decompose the group ERPs into latent components but also to assess the reliability of components. In particular, the optimal number of latent components is determined by the BIC (MacKay, 1992; Hansen et al., 2001; Kolenda et al., 2001), while the split-half reliability of the model is estimated by repeating the procedure 1000 times for a randomly selected half of the total ERP set. Although the reliability of the components presented in this article is very high, the given method still remains one of many other similar approaches.

The other limitation of the study is the application of sLORETA for localization of the sources of latent components. Basically a component is uniquely characterized by its topography so that the associated sLORETA image, as a linear transformation of the topography, does not provide any new information and has been used in the current study for a rough approximation of source localization of components. We hope that this information could be further used in future imaging studies with the goal to associate operations of cognitive control with neuronal networks of the human brain.

And finally, this is a descriptive study. We know little about the functional meaning of the components and their parts and can only speculate based on a loose association with previous studies of ERP waves. The first way to study the functional meaning of the components would be to manipulate the stimulus properties and task conditions. Initial attempts in this direction have been made by Kropotov et al. (2011). The second way would be to correlate the latent components with measures from neuropsychological tests, similarly to what has been done by Brunner et al. (2015).

## Conclusion

As people age, their performance on tasks requiring cognitive control largely declines. There are two general hypotheses of aging used to explain this decline: the general resource and specific process hypotheses. In the general resource hypothesis, it is argued that the decline in cognitive control depends mainly on a general age-related decline in cognitive control capacity, while the specific process hypothesis suggests age-related declines in specific cognitive functions. In order to test which of these hypotheses best explain age-related changes in cognitive control, ERP correlates of proactive cognitive control were studied in a large group of healthy subjects between 18 and 84 years of age.

The subjects performed a relatively demanding cued GO/NOGO with age differences in terms of omissions (the older the less) and reaction times (the older the slower). The ERP waves of cognitive control—N2, P3cue and CNV—were decomposed into latent components by means of a blind source separation method (Kropotov and Ponomarev, 2015). The processing speed hypothesis (Salthouse, 1996) was supported by the finding that the latencies of all cognitive control components increase with age: early components (<200 ms) increase at 8 ms per decade while late components increase at 20 ms per decade. The compensatory hypothesis of aging (Park and Reuter-Lorenz, 2009) was supported by the observation that the amplitudes of the components localized in the posterior areas decrease with age, while the amplitudes of the components localized in the prefrontal cortical areas increase with age in order to maintain performance on this simple task at a relatively stable level.

## Author Contributions

YJK: worked on the manuscript, did the data analysis, interpreted the data, drafted the manuscript. VP: worked on the manuscript, did the data analysis. EPT: did the data analysis. AM: measured most of the older subjects. LJ: worked on the manuscript, interpreted the data, drafted the manuscript.

## Conflict of Interest Statement

The authors declare that the research was conducted in the absence of any commercial or financial relationships that could be construed as a potential conflict of interest.
